# Crystal structure of bis­[μ-bis­(di­phenyl­phosphanyl)methane-κ^2^ *P*:*P*′]digold(I) dichloride acetone monosolvate monohydrate

**DOI:** 10.1107/S2056989015013341

**Published:** 2015-07-17

**Authors:** Chien Ing Yeo, Yee Seng Tan, Edward R. T. Tiekink

**Affiliations:** a Department of Chemistry, University of Malaya, 50603 Kuala Lumpur, Malaysia

**Keywords:** crystal structure, phosphanegold(I) salt, pseudopolymorph, aurophilic inter­action, hydrogen bonding

## Abstract

In the title complex salt, [Au_2_{(C_6_H_5_)_2_PCH_2_P(C_6_H_5_)_2_}]Cl_2_·(CH_3_)_2_C=O·H_2_O, the dication forms an eight-membered {—PCPAu}_2_ ring with a transannular aurophilic inter­action [Au⋯Au = 2.9743 (2) Å]. The ring approximates a flattened boat conformation, with the two methyl­ene C atoms lying *ca* 0.58–0.59 Å above the least-squares plane defined by the Au_2_P_4_ atoms (r.m.s. deviation = 0.0849 Å). One Cl^−^ anion functions as a weak bridge between the Au^I^ atoms [Au⋯Cl = 2.9492 (13) and 2.9776 (12) Å]. The second Cl^−^ anion forms two (water)O—H⋯Cl hydrogen bonds about a centre of inversion, forming a centrosymmetric eight-membered {⋯HOH⋯Cl}_2_ supra­molecular square. Globally, the dications and loosely associated Cl^−^ anions assemble into layers lying parallel to the *ac* plane, being connected by C—H⋯Cl,π(phen­yl) inter­actions. The supra­molecular squares and solvent acetone mol­ecules are sandwiched in the inter-layer region, being connected to the layers on either side by C—H⋯Cl,O(acetone) inter­actions.

## Abstract 2

The Au^I^ atoms in the dication of the title structure, [(C_6_H_5_)_2_PCH_2_P(C_6_H_5_)_2_Au_2_]Cl_2_·(CH_3_)_2_C=O·H_2_O, show an aurophilic inter­action of 2.9743 (2) Å.

## Chemical context   

Recent studies have highlighted the significant biological activity exhibited by phosphanegold(I) carbonimido­thio­ates, *i.e*. compounds of the general formula Ph_3_PAu[SC(O*R*)=N(ar­yl)]; *R* = alkyl. These compounds are cytotoxic and kill cancer cells by initiating apoptotic pathways (Yeo, Ooi *et al.*, 2013 ▸; Ooi *et al.*, 2015 ▸) and prove to be very potent to Gram-positive bacteria (Yeo, Sim *et al.*, 2013 ▸). Over and above this potential, phosphanegold(I) carbonimido­thio­ates offer opportunities in crystal engineering (Kuan *et al.*, 2008 ▸) and exhibit solid-state luminescence (Ho *et al.*, 2006 ▸). 
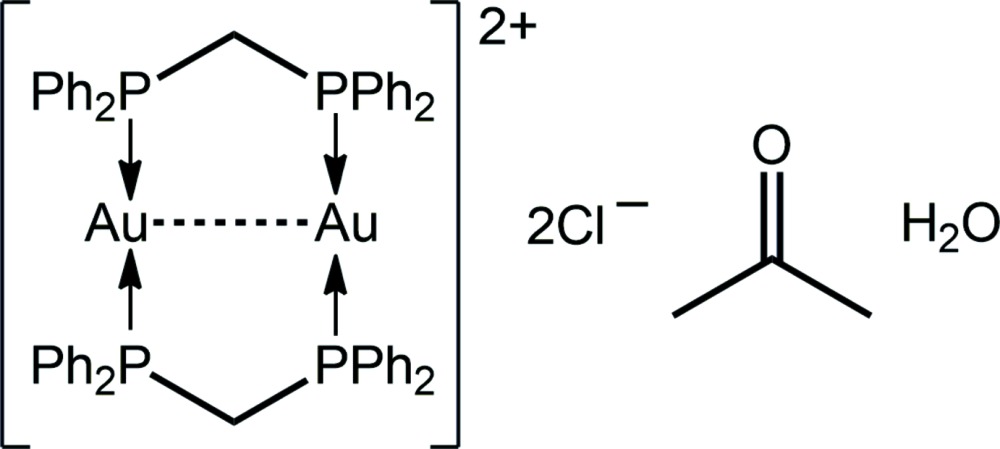


As a part of an effort to increase the nuclearity of these phosphanegold(I) thiol­ates, reactions with the bipodal mol­ecule, {1,4-[MeOC(=S)N(H)]_2_C_6_H_4_} (Yeo *et al.*, 2015 ▸), were performed. When the bridging phosphane ligand was bis­(di­phenyl­phosphane)methane, the title salt, [Au_2_(Ph_2_PCH_2_PPh_2_)]Cl_2_, was isolated as an acetone monosolvate monohydrate, (I). The structure of (I) is discussed herein along with a comparison with analogous [Au_2_(Ph_2_PCH_2_PPh_2_)]Cl_2_ salts characterized as an acetone solvate (Schmidbaur *et al.*, 1977 ▸) and as an aceto­nitrile solvate (Liou *et al.*, 1994 ▸), as well as related species.

## Structural commentary   

**Figure 1:**
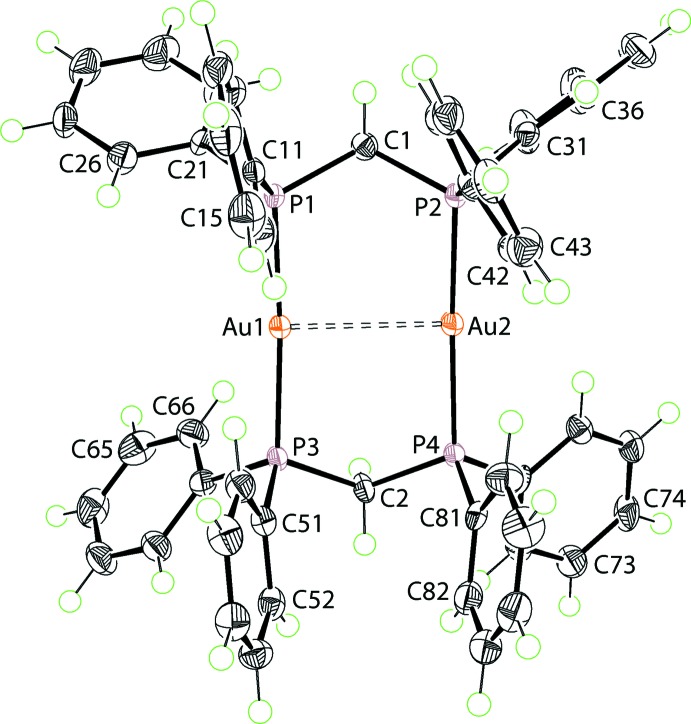
The mol­ecular structure of the [Au_2_(Ph_2_PCH_2_PPh_2_)]^2+^ dication in (I), showing the atom-labelling scheme and displacement ellipsoids at the 70% probability level.

The asymmetric unit of (I) comprises a [Au_2_(Ph_2_PCH_2_PPh_2_)]^2+^ dication, two Cl^−^ anions, and a solvent mol­ecule each of acetone and water; all species are in general positions. The mol­ecular structure of the dication is shown in Fig. 1 ▸. Two Au^I^ atoms are bridged by two Ph_2_PCH_2_PPh_2_ ligands, forming an eight-membered {—PCPAu}_2_ ring. The ring has the form of a boat with the methyl­ene-C1 and C2 atoms lying to one side of the ring and 0.589 (5) and 0.581 (5) Å, respectively, above the least-squares plane through the Au_2_P_4_ atoms which have a r.m.s. deviation of 0.0849 Å. There is a transannular Au1⋯Au2 (aurophilic) inter­action of 2.9743 (2) Å. This inter­action is partly responsible for the deviations of the P1—Au1—P3 and P2—Au2—P4 angles from the ideal 180°, *i.e*. 173.24 (4) and 170.04 (4)°, respectively. The Au—P bond lengths are almost equivalent, ranging from a short Au1—P1 2.3061 (12) to a long Au2—P4 2.3130 (12) Å. The Cl1^−^ anion forms a weak bridge between the two Au^I^ atoms with Au1⋯Cl1 and Au2⋯Cl2 separations of 2.9492 (13) and 2.9776 (12) Å, respectively. The second Cl^−^ anion participates in hydrogen bonding as described below in *Supra­molecular features*.

## Supra­molecular features   

**Figure 2:**
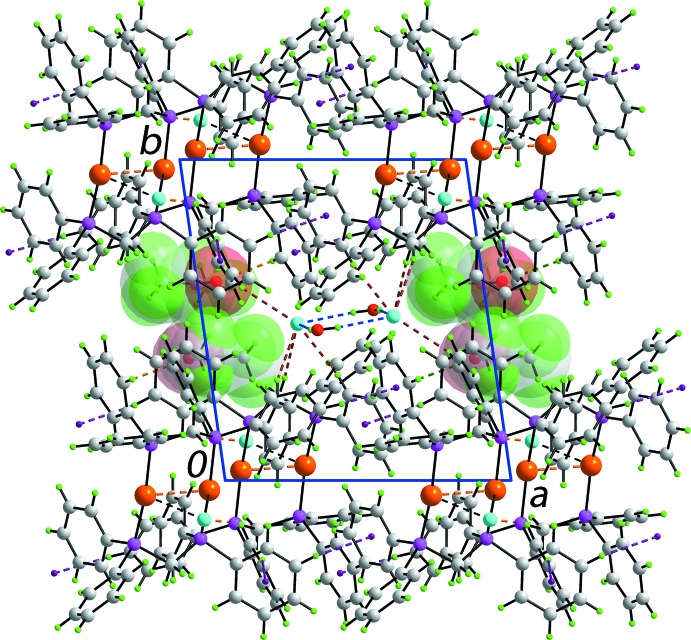
Unit-cell contents of (I) shown in projection down the *c* axis. Intra­molecular aurophilic inter­actions are drawn as orange dashed lines and the weak Au⋯Cl contacts are shown as black dashed lines. Inter­molecular O—H⋯Cl, C—H⋯Cl1, C—H⋯Cl2, C—H⋯O(acetone) and C—H⋯π inter­actions are shown as blue, orange, brown, green and purple dashed lines, respectively. The acetone mol­ecules have been highlighted in space-filling mode.

The most notable feature of the crystal packing of (I) is the formation of (water)O—H⋯Cl2 hydrogen bonds that lead to centrosymmetric eight-membered {⋯HOH⋯Cl}_2_ supra­molecular squares with edge lengths of 3.217 (5) and 3.200 (5) Å, Table 1 ▸ (Spek, 2009 ▸). These reside parallel to the *ac* plane, corresponding to the inter-layer region between layers of dications and Cl1^−^ anions, Fig. 2 ▸. Three independent (phen­yl)C—H⋯π(phen­yl) contacts occur between the dicat­ions. The Cl1^−^ anion forms a single (phen­yl)C—H⋯Cl contact, a reduced propensity reflecting its close association with the Au^I^ atoms (see above). By contrast, the Cl2^−^ anion forms four independent C—H⋯Cl2 inter­actions, *i.e*. a (methyl­ene)C—H⋯Cl2 and three (phen­yl)C—H⋯Cl2 inter­actions, providing links between the {⋯HOH⋯Cl}_2_ rings and the cations. The acetone solvent mol­ecule accepts a (methyl­ene)- and a (phen­yl)C—H⋯O contact.

## Database survey   

**Figure 3:**
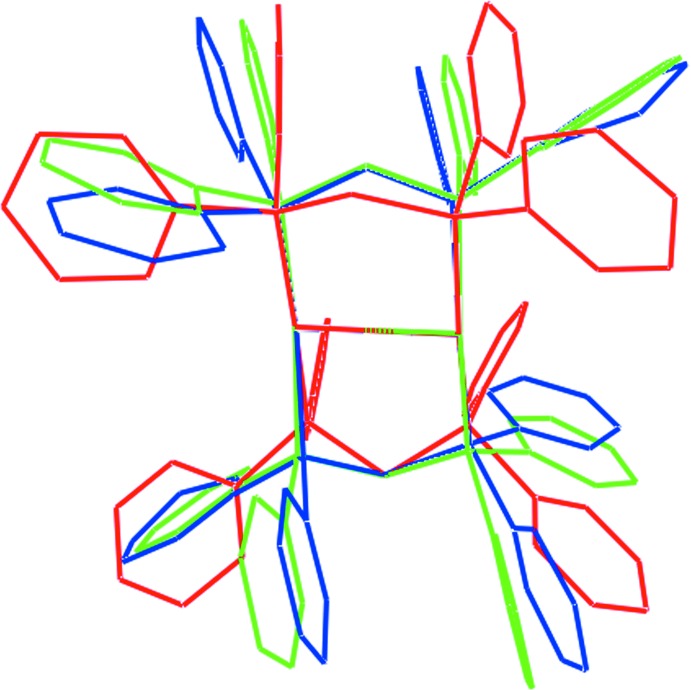
Overlay diagram of the [Au_2_(Ph_2_PCH_2_PPh_2_)]^2+^ dications in (I) (red image), LEKGAJ (green) and PPEAUC (blue), overlapped so that the one methyl­ene C and the two Au^I^ atoms are coincident.

**Figure 4:**
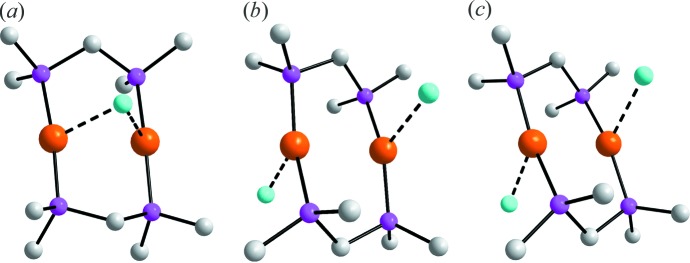
Details of the weak Au⋯Cl inter­actions, shown as dashed black lines, in the dications of (*a*) (I), (*b*) LEKGAJ and (*c*) PPEAUC. For clarity, all H atoms have been removed and only the *ipso*-C atoms shown.

The [Au_2_(Ph_2_PCH_2_PPh_2_)]Cl_2_ salt has been characterized twice previously, originally as an acetone solvate (Schmidbaur *et al.*, 1977 ▸) and subsequently as an aceto­nitrile solvate (Liou *et al.*, 1994 ▸). Geometric data characterizing the eight-membered rings are summarized in Table 2 ▸. The most notable difference between the structure of (I) and the dications is that the latter are disposed about a centre of inversion and the eight-membered {—PCPAu}_2_ rings have flattened chair conformations, with the methyl­ene-C atoms lying to either side of the eight-membered ring. The similarity between the literature structures and the difference between these and the dication in (I) are highlighted in the overlay diagram shown in Fig. 3 ▸. The other remarkable difference between the three structur­ally characterized [Au_2_(Ph_2_PCH_2_PPh_2_)]Cl_2_ salts relates to the mode of association between the complex Au cations and Cl^−^ anions. As noted above and shown in Fig. 4 ▸ *a*, the Cl1^−^ anion in (I) forms a weak bridge between the two Au^I^ atoms. In the acetone solvate (Schmidbaur *et al.*, 1977 ▸), each Cl^−^ anion associates with one Au^I^ atom at a distance of 2.771 (4) Å. A similar pattern is noted in the aceto­nitrile solvate (Liou *et al.*, 1994 ▸), but the distances are significantly longer at 2.951 (4) Å. The close Au⋯Cl contacts appear to influence the P—Au—P angles in that those in the [Au_2_(Ph_2_PCH_2_PPh_2_)]Cl_2_ salts with loosely associated Cl^−^ anions having greater distortions from linearity, in particular for the acetone solvate (Schmidbaur *et al.*, 1977 ▸), compared with dications characterized with non-coordinating counter-anions, namely BH_4_^−^ (Porter *et al.*, 1989 ▸), ClO_4_^−^ (Cao *et al.*, 2006 ▸), PF_6_^−^ (Wu *et al.*, 2003 ▸) and [H_3_BCN]^−^ (Khan *et al.*, 1989 ▸), Table 2 ▸.

## Synthesis and crystallization   

The title compound is an unexpected product from the reaction of bis­[chlorido­gold(I)] bis­(di­phenyl­phosphane)methane with an equimolar amount of {1,4-[MeOC(=S)N(H)]_2_C_6_H_4_} (Yeo *et al.*, 2015 ▸). The preparation was as follows. To the gold precursor, (Ph_2_PCH_2_PPh_2_)(AuCl)_2_ (0.5 mmol, 0.42 g) in aceto­nitrile (50 ml) was added NaOH (1.0 mmol, 0.04 g in 20 ml H_2_O) and {1,4-[MeOC(=S)N(H)]_2_C_6_H_4_} (0.5 mmol, 0.13 g) in aceto­nitrile (50 ml). The resulting mixture was stirred at 323 K for 2 h. The final product was extracted with di­chloro­methane (100 ml) and the solution was left for evaporation at room temperature. After 3 weeks a slurry formed. This was redissolved in a solvent mixture of acetone/aceto­nitrile (1:1 *v*/*v*, 100 ml) and left for slow evaporation. Colourless crystals were obtained after 10 days. Yield: 0.213 g (43%). M.p. 477–479 K. ^1^H NMR (400 MHz, acetone-*d*6, 298 K): δ 7.96 (*d*, 8H, *o*-Ph-H, *J* = 6.20 Hz), 7.49 (*t*, 4H, *p*-Ph-H, *J* = 7.32 Hz), 7.41 (*t*, 8H, *m*-Ph-H, *J* = 7.82 Hz), 4.84 (*s*, *br*, 2H, CH_2_), 2.82 (*s*, *br*, 1H, H_2_O). Analysis calculated for C_53_H_52_Au_2_Cl_2_O_2_P_4_: C, 48.61; H, 4.00. Found: C, 48.64; H, 3.99. IR (cm^−1^): 3044 (*m*) ν(C—H), 1484 (*s*) ν(C—C).

## Refinement   

Crystal data, data collection and structure refinement details are summarized in Table 3 ▸. Carbon-bound H-atoms were placed in calculated positions (C—H = 0.95–0.99 Å) and were included in the refinement in the riding-model approximation, with *U*_iso_(H) set to 1.2–1.5*U*_equiv_(C). The water-bound H atoms were refined with O—H = 0.84±0.01 Å, and with *U*_iso_(H) = 1.5*U*_equiv_(O). The *U*_33_ parameter was elongated for the C93 atom. In the final refinement this was restrained to be nearly isotropic using the ISOR command in *SHELXL* (Sheldrick, 2015 ▸). The maximum and minimum residual electron density peaks of 3.50 and 1.82 eÅ^−3^, respectively, were located 0.90 Å and 0.78 Å from the Au1 and Au2 atoms, respectively.

## Supplementary Material

Crystal structure: contains datablock(s) I, global. DOI: 10.1107/S2056989015013341/wm5185sup1.cif

Structure factors: contains datablock(s) I. DOI: 10.1107/S2056989015013341/wm5185Isup2.hkl

CCDC reference: 1412185

Additional supporting information:  crystallographic information; 3D view; checkCIF report

## Figures and Tables

**Table 1:** Hydrogen-bond geometry (, ) *Cg*1*Cg*3 are the ring centroids of the C11C16, C71C76 and C51C56 benzene rings, respectively.

*D*H*A*	*D*H	H*A*	*D* *A*	*D*H*A*
O1*W*H1*W*Cl2	0.84(2)	2.39(2)	3.217(5)	168(6)
O1*W*H2*W*Cl2^i^	0.84(3)	2.37(3)	3.200(5)	173(3)
C1H1*B*O1^ii^	0.99	2.35	3.310(7)	164
C2H2*B*Cl2^iii^	0.99	2.51	3.487(5)	168
C12H12O1^ii^	0.95	2.57	3.249(7)	129
C22H22Cl1^ii^	0.95	2.74	3.580(6)	148
C44H44Cl2^iv^	0.95	2.73	3.424(5)	130
C52H52Cl2^iii^	0.95	2.68	3.616(5)	169
C82H82Cl2^iii^	0.95	2.81	3.723(5)	161
C34H34*Cg*1^v^	0.95	2.82	3.542(6)	133
C43H43*Cg*2^vi^	0.95	2.74	3.574(5)	147
C75H75*Cg*3^v^	0.95	2.83	3.619(5)	142

Symmetry codes: (i) 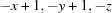; (ii) 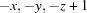; (iii) 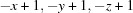; (iv) 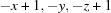; (v) 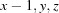; (vi) 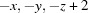.

**Table 2:** Summary of [Au_2_(Ph_2_PCH_2_PPh_2_)]^2+^ dication structures

Anion	solvent	symmetry	AuAu	AuP	PAuP	CCDC REFCODE	Reference
Cl	Me_2_CO	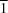	2.962(1)	2.327(3), 2.288(3)	155.9(1)	PPEAUC	Schmidbaur *et al.* (1977 ▸)
Cl	MeCN	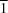	2.9941(8)	2.333(3), 2.299(3)	164.90(9)	LEKGAJ	Liou *et al.* (1994 ▸)
Cl	Me_2_CO, H_2_O	1	2.9743(2)	2.3061(12), 2.3102(12); 2.3082(12), 2.3130(12)	173.24(4); 170.04(4)		this work
BH_4_		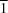	2.931(1)	2.311(3), 2.310(3)	177.28(12)	JAMKAJ	Porter *et al.* (1989 ▸)
ClO_4_		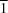	2.9258(10)	2.3118(15), 2.3139(15)	177.15(5)	NEQNIH	Cao *et al.* (2006 ▸)
PF_6_	CH_2_Cl_2_	2	2.9792(10)	2.314(3), 2.318(3)	177.85(13)	MUVVEE	Wu *et al.* (2003 ▸)
H_3_BCN	CH_2_Cl_2_	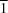	2.982(3)	2.311(6), 2.329(6)	175.2(2)	SAVRAI	Khan *et al.* (1989 ▸)

Note: (*a*) Groom Allen (2014 ▸).

**Table 3:** Experimental details

Crystal data	
Chemical formula	[Au_2_(C_25_H_22_P_2_)_2_]Cl_2_C_3_H_6_OH_2_O
*M*_r_	1309.66
Crystal system, space group	Triclinic, *P* 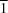
Temperature (K)	100
*a*, *b*, *c* ()	11.7708(3), 13.3657(3), 16.1209(4)
, , ()	94.056(2), 92.059(2), 97.882(2)
*V* (^3^)	2503.29(11)
*Z*	2
Radiation type	Mo *K*
(mm^1^)	6.13
Crystal size (mm)	0.22 0.12 0.07

Data collection	
Diffractometer	Agilent SuperNova Dual diffractometer with an Atlas detector
Absorption correction	Multi-scan (*CrysAlis PRO*; Agilent, 2013 ▸)
*T*_min_, *T*_max_	0.544, 1.000
No. of measured, independent and observed [*I* > 2(*I*)] reflections	56582, 11486, 9744
*R*_int_	0.069
(sin /)_max_ (^1^)	0.650

Refinement	
*R*[*F* ^2^ > 2(*F* ^2^)], *wR*(*F* ^2^), *S*	0.037, 0.104, 1.10
No. of reflections	11486
No. of parameters	576
No. of restraints	9
H-atom treatment	H atoms treated by a mixture of independent and constrained refinement
_max_, _min_ (e ^3^)	3.50, 1.82

Computer programs: *CrysAlis PRO* (Agilent, 2013 ▸), *SHELXS97* (Sheldrick, 2008 ▸), *SHELXL2014* (Sheldrick, 2015 ▸), *ORTEP-3 for Windows* (Farrugia, 2012 ▸), *QMol* (Gans Shalloway, 2001 ▸), *DIAMOND* (Brandenburg, 2006 ▸) and *publCIF* (Westrip, 2010 ▸).

## supplementary crystallographic information

### Crystal data

**Table:**

[Au_2_(C_25_H_22_P_2_)_2_]Cl_2_·C_3_H_6_O·H_2_O	*Z* = 2
*M**_r_* = 1309.66	*F*(000) = 1276
Triclinic, *P*1	*D*_x_ = 1.737 Mg m^−^^3^
*a* = 11.7708 (3) Å	Mo *K*α radiation, λ = 0.71073 Å
*b* = 13.3657 (3) Å	Cell parameters from 22677 reflections
*c* = 16.1209 (4) Å	θ = 3.0–30.2°
α = 94.056 (2)°	µ = 6.13 mm^−^^1^
β = 92.059 (2)°	*T* = 100 K
γ = 97.882 (2)°	Prism, colourless
*V* = 2503.29 (11) Å^3^	0.22 × 0.12 × 0.07 mm

### Data collection

**Table:**

Agilent SuperNova Dual diffractometer with an Atlas detector	11486 independent reflections
Radiation source: Agilent SuperNova (Mo) X-ray Source	9744 reflections with *I* > 2σ(*I*)
Mirror monochromator	*R*_int_ = 0.069
Detector resolution: 10.4041 pixels mm^-1^	θ_max_ = 27.5°, θ_min_ = 2.9°
ω scan	*h* = −15→15
Absorption correction: multi-scan (*CrysAlis PRO*; Agilent, 2013)	*k* = −17→17
*T*_min_ = 0.544, *T*_max_ = 1.000	*l* = −20→20
56582 measured reflections	

### Refinement

**Table:**

Refinement on *F*^2^	9 restraints
Least-squares matrix: full	Hydrogen site location: mixed
*R*[*F*^2^ > 2σ(*F*^2^)] = 0.037	H atoms treated by a mixture of independent and constrained refinement
*wR*(*F*^2^) = 0.104	*w* = 1/[σ^2^(*F*_o_^2^) + (0.0555*P*)^2^ + 0.3597*P*] where *P* = (*F*_o_^2^ + 2*F*_c_^2^)/3
*S* = 1.10	(Δ/σ)_max_ = 0.003
11486 reflections	Δρ_max_ = 3.50 e Å^−^^3^
576 parameters	Δρ_min_ = −1.81 e Å^−^^3^

### Special details

**Table:**

Geometry. All e.s.d.'s (except the e.s.d. in the dihedral angle between two l.s. planes) are estimated using the full covariance matrix. The cell e.s.d.'s are taken into account individually in the estimation of e.s.d.'s in distances, angles and torsion angles; correlations between e.s.d.'s in cell parameters are only used when they are defined by crystal symmetry. An approximate (isotropic) treatment of cell e.s.d.'s is used for estimating e.s.d.'s involving l.s. planes.
Refinement. The maximum and minimum residual electron density peaks of 3.50 and 1.82 e Å^-3^, respectively, were located 0.90 Å and 0.78 Å from the Au1 and Au2 atoms, respectively.

### Fractional atomic coordinates and isotropic or equivalent isotropic displacement parameters (Å^2^)

**Table:**

	*x*	*y*	*z*	*U*_iso_*/*U*_eq_	
Au1	0.28677 (2)	0.04681 (2)	0.68901 (2)	0.01293 (6)	
Au2	0.06008 (2)	0.03012 (2)	0.76651 (2)	0.01298 (6)	
Cl1	0.09244 (11)	0.12210 (10)	0.60460 (7)	0.0240 (3)	
Cl2	0.66940 (11)	0.51164 (9)	0.08410 (8)	0.0240 (3)	
P1	0.24235 (10)	−0.11146 (9)	0.61967 (7)	0.0113 (2)	
P2	0.01377 (10)	−0.13401 (9)	0.70857 (7)	0.0122 (2)	
P3	0.35008 (10)	0.19848 (9)	0.76508 (7)	0.0120 (2)	
P4	0.11500 (10)	0.18167 (9)	0.84524 (7)	0.0117 (2)	
O1	−0.0662 (4)	0.3808 (3)	0.4856 (3)	0.0386 (10)	
O1W	0.3973 (4)	0.4688 (3)	0.1077 (3)	0.0345 (9)	
H1W	0.4694 (10)	0.477 (5)	0.109 (4)	0.052*	
H2W	0.375 (5)	0.469 (5)	0.0576 (14)	0.052*	
C1	0.0875 (4)	−0.1522 (4)	0.6120 (3)	0.0146 (9)	
H1A	0.0531	−0.1149	0.5689	0.017*	
H1B	0.0736	−0.2251	0.5930	0.017*	
C2	0.2293 (4)	0.2626 (4)	0.7984 (3)	0.0147 (10)	
H2A	0.1961	0.2910	0.7494	0.018*	
H2B	0.2590	0.3200	0.8391	0.018*	
C11	0.3081 (4)	−0.2114 (3)	0.6638 (3)	0.0140 (9)	
C12	0.3073 (4)	−0.3048 (4)	0.6191 (3)	0.0188 (10)	
H12	0.2748	−0.3154	0.5639	0.023*	
C13	0.3539 (4)	−0.3820 (4)	0.6548 (3)	0.0224 (11)	
H13	0.3527	−0.4455	0.6242	0.027*	
C14	0.4020 (5)	−0.3671 (4)	0.7348 (3)	0.0254 (12)	
H14	0.4326	−0.4208	0.7593	0.031*	
C15	0.4058 (5)	−0.2736 (4)	0.7799 (3)	0.0268 (12)	
H15	0.4404	−0.2628	0.8346	0.032*	
C16	0.3588 (4)	−0.1968 (4)	0.7442 (3)	0.0199 (10)	
H16	0.3610	−0.1331	0.7747	0.024*	
C21	0.2820 (4)	−0.1187 (3)	0.5120 (3)	0.0133 (9)	
C22	0.2038 (5)	−0.1342 (4)	0.4457 (3)	0.0224 (11)	
H22	0.1239	−0.1416	0.4549	0.027*	
C23	0.2417 (5)	−0.1391 (4)	0.3650 (3)	0.0294 (13)	
H23	0.1871	−0.1499	0.3192	0.035*	
C24	0.3569 (5)	−0.1286 (4)	0.3505 (3)	0.0287 (12)	
H24	0.3819	−0.1322	0.2951	0.034*	
C25	0.4365 (5)	−0.1126 (4)	0.4174 (3)	0.0225 (11)	
H25	0.5163	−0.1049	0.4077	0.027*	
C26	0.4001 (4)	−0.1079 (4)	0.4978 (3)	0.0197 (10)	
H26	0.4547	−0.0975	0.5435	0.024*	
C31	−0.1349 (4)	−0.1735 (4)	0.6745 (3)	0.0154 (10)	
C32	−0.1851 (5)	−0.1157 (4)	0.6191 (3)	0.0284 (12)	
H32	−0.1403	−0.0595	0.5977	0.034*	
C33	−0.2992 (5)	−0.1402 (4)	0.5945 (3)	0.0304 (13)	
H33	−0.3328	−0.1008	0.5561	0.036*	
C34	−0.3661 (5)	−0.2226 (4)	0.6255 (3)	0.0295 (12)	
H34	−0.4453	−0.2386	0.6095	0.035*	
C35	−0.3150 (5)	−0.2803 (4)	0.6798 (3)	0.0272 (12)	
H35	−0.3598	−0.3370	0.7004	0.033*	
C36	−0.1999 (4)	−0.2572 (4)	0.7047 (3)	0.0198 (10)	
H36	−0.1658	−0.2978	0.7419	0.024*	
C41	0.0521 (4)	−0.2259 (4)	0.7777 (3)	0.0158 (10)	
C42	0.0559 (4)	−0.2008 (4)	0.8632 (3)	0.0176 (10)	
H42	0.0342	−0.1381	0.8836	0.021*	
C43	0.0906 (5)	−0.2656 (4)	0.9184 (3)	0.0233 (11)	
H43	0.0905	−0.2483	0.9766	0.028*	
C44	0.1259 (4)	−0.3563 (4)	0.8895 (3)	0.0217 (11)	
H44	0.1538	−0.3994	0.9275	0.026*	
C45	0.1200 (4)	−0.3833 (4)	0.8043 (3)	0.0222 (11)	
H45	0.1421	−0.4460	0.7842	0.027*	
C46	0.0824 (4)	−0.3200 (4)	0.7489 (3)	0.0191 (10)	
H46	0.0770	−0.3398	0.6910	0.023*	
C51	0.4417 (4)	0.1860 (4)	0.8555 (3)	0.0165 (10)	
C52	0.4751 (4)	0.2664 (4)	0.9156 (3)	0.0168 (10)	
H52	0.4455	0.3285	0.9113	0.020*	
C53	0.5503 (4)	0.2553 (4)	0.9814 (3)	0.0191 (10)	
H53	0.5742	0.3106	1.0212	0.023*	
C54	0.5908 (4)	0.1638 (4)	0.9891 (3)	0.0209 (11)	
H54	0.6416	0.1561	1.0346	0.025*	
C55	0.5569 (5)	0.0832 (4)	0.9300 (3)	0.0220 (11)	
H55	0.5842	0.0202	0.9356	0.026*	
C56	0.4834 (4)	0.0942 (4)	0.8632 (3)	0.0188 (10)	
H56	0.4615	0.0392	0.8226	0.023*	
C61	0.4295 (4)	0.2909 (3)	0.7029 (3)	0.0166 (10)	
C62	0.5306 (4)	0.3522 (4)	0.7314 (3)	0.0203 (11)	
H62	0.5591	0.3493	0.7869	0.024*	
C63	0.5894 (5)	0.4169 (4)	0.6803 (3)	0.0252 (11)	
H63	0.6582	0.4584	0.7004	0.030*	
C64	0.5480 (5)	0.4215 (4)	0.5988 (3)	0.0274 (12)	
H64	0.5890	0.4657	0.5631	0.033*	
C65	0.4484 (5)	0.3624 (4)	0.5702 (3)	0.0269 (12)	
H65	0.4200	0.3664	0.5148	0.032*	
C66	0.3879 (5)	0.2959 (4)	0.6218 (3)	0.0220 (11)	
H66	0.3190	0.2547	0.6016	0.026*	
C71	0.0015 (4)	0.2598 (3)	0.8562 (3)	0.0130 (9)	
C72	0.0215 (4)	0.3630 (4)	0.8813 (3)	0.0197 (10)	
H72	0.0979	0.3965	0.8909	0.024*	
C73	−0.0707 (5)	0.4167 (4)	0.8922 (3)	0.0235 (11)	
H73	−0.0567	0.4867	0.9102	0.028*	
C74	−0.1822 (4)	0.3699 (4)	0.8772 (3)	0.0235 (11)	
H74	−0.2445	0.4075	0.8846	0.028*	
C75	−0.2031 (4)	0.2672 (4)	0.8514 (3)	0.0225 (11)	
H75	−0.2797	0.2348	0.8407	0.027*	
C76	−0.1127 (4)	0.2124 (4)	0.8411 (3)	0.0162 (10)	
H76	−0.1276	0.1422	0.8237	0.019*	
C81	0.1678 (4)	0.1642 (4)	0.9500 (3)	0.0147 (9)	
C82	0.2221 (4)	0.2446 (4)	1.0032 (3)	0.0202 (11)	
H82	0.2317	0.3112	0.9850	0.024*	
C83	0.2624 (5)	0.2283 (4)	1.0825 (3)	0.0235 (11)	
H83	0.3009	0.2831	1.1181	0.028*	
C84	0.2458 (5)	0.1309 (4)	1.1091 (3)	0.0238 (11)	
H84	0.2724	0.1191	1.1635	0.029*	
C85	0.1914 (5)	0.0519 (4)	1.0576 (3)	0.0281 (12)	
H85	0.1807	−0.0143	1.0765	0.034*	
C86	0.1515 (5)	0.0671 (4)	0.9777 (3)	0.0225 (11)	
H86	0.1136	0.0117	0.9424	0.027*	
C91	0.0033 (6)	0.3868 (4)	0.5431 (4)	0.0430 (17)	
C92	−0.0315 (11)	0.3930 (7)	0.6302 (5)	0.098 (4)	
H92A	−0.1130	0.4009	0.6313	0.117*	
H92B	0.0145	0.4514	0.6611	0.117*	
H92C	−0.0190	0.3309	0.6559	0.117*	
C93	0.1267 (7)	0.3843 (6)	0.5275 (7)	0.086 (3)	
H93A	0.1430	0.4069	0.4721	0.130*	
H93B	0.1443	0.3151	0.5305	0.130*	
H93C	0.1742	0.4295	0.5696	0.130*	

### Atomic displacement parameters (Å^2^)

**Table:**

	*U*^11^	*U*^22^	*U*^33^	*U*^12^	*U*^13^	*U*^23^
Au1	0.01005 (10)	0.01270 (11)	0.01551 (10)	0.00047 (7)	0.00282 (7)	−0.00157 (7)
Au2	0.01054 (10)	0.01158 (11)	0.01655 (11)	0.00107 (7)	0.00271 (7)	−0.00093 (7)
Cl1	0.0188 (6)	0.0282 (7)	0.0257 (6)	0.0037 (5)	−0.0032 (5)	0.0079 (5)
Cl2	0.0206 (7)	0.0165 (6)	0.0345 (7)	0.0041 (5)	−0.0052 (5)	−0.0009 (5)
P1	0.0081 (6)	0.0128 (6)	0.0130 (6)	0.0020 (5)	0.0017 (4)	−0.0010 (4)
P2	0.0093 (6)	0.0118 (6)	0.0152 (6)	0.0010 (5)	0.0028 (4)	−0.0005 (4)
P3	0.0087 (6)	0.0117 (6)	0.0153 (6)	0.0009 (5)	0.0023 (4)	0.0000 (5)
P4	0.0103 (6)	0.0111 (6)	0.0138 (6)	0.0010 (5)	0.0026 (4)	0.0005 (4)
O1	0.040 (3)	0.028 (2)	0.045 (3)	0.0041 (19)	−0.017 (2)	−0.0045 (19)
O1W	0.025 (2)	0.038 (2)	0.036 (2)	−0.004 (2)	−0.0014 (18)	−0.0037 (19)
C1	0.013 (2)	0.016 (2)	0.015 (2)	0.0026 (19)	−0.0002 (17)	0.0010 (18)
C2	0.010 (2)	0.014 (2)	0.021 (2)	0.0006 (18)	0.0032 (17)	0.0026 (19)
C11	0.010 (2)	0.013 (2)	0.019 (2)	0.0016 (18)	0.0039 (17)	0.0012 (18)
C12	0.013 (2)	0.022 (3)	0.021 (2)	0.004 (2)	0.0017 (18)	−0.003 (2)
C13	0.019 (3)	0.017 (3)	0.032 (3)	0.004 (2)	0.010 (2)	0.001 (2)
C14	0.018 (3)	0.028 (3)	0.033 (3)	0.007 (2)	0.007 (2)	0.014 (2)
C15	0.024 (3)	0.039 (3)	0.020 (3)	0.006 (2)	0.001 (2)	0.008 (2)
C16	0.016 (3)	0.027 (3)	0.017 (2)	0.003 (2)	0.0044 (18)	0.005 (2)
C21	0.012 (2)	0.014 (2)	0.014 (2)	0.0021 (18)	0.0048 (17)	−0.0011 (17)
C22	0.023 (3)	0.027 (3)	0.018 (2)	0.006 (2)	0.007 (2)	0.003 (2)
C23	0.032 (3)	0.039 (3)	0.016 (3)	0.005 (3)	0.000 (2)	−0.004 (2)
C24	0.034 (3)	0.033 (3)	0.019 (3)	0.003 (3)	0.012 (2)	0.002 (2)
C25	0.018 (3)	0.022 (3)	0.028 (3)	0.002 (2)	0.011 (2)	0.002 (2)
C26	0.019 (3)	0.023 (3)	0.018 (2)	0.005 (2)	0.0015 (19)	0.001 (2)
C31	0.010 (2)	0.017 (2)	0.019 (2)	0.0027 (19)	0.0011 (17)	−0.0055 (19)
C32	0.018 (3)	0.030 (3)	0.039 (3)	0.005 (2)	0.006 (2)	0.011 (2)
C33	0.014 (3)	0.041 (3)	0.038 (3)	0.009 (2)	−0.002 (2)	0.011 (3)
C34	0.012 (3)	0.036 (3)	0.039 (3)	0.002 (2)	0.000 (2)	−0.006 (3)
C35	0.017 (3)	0.021 (3)	0.041 (3)	−0.002 (2)	0.002 (2)	−0.001 (2)
C36	0.013 (3)	0.020 (3)	0.027 (3)	0.000 (2)	0.0036 (19)	0.001 (2)
C41	0.009 (2)	0.017 (2)	0.020 (2)	0.0005 (19)	−0.0001 (17)	0.0020 (19)
C42	0.016 (3)	0.015 (2)	0.021 (2)	0.0012 (19)	0.0016 (18)	−0.0006 (19)
C43	0.024 (3)	0.022 (3)	0.024 (3)	0.004 (2)	0.004 (2)	0.004 (2)
C44	0.015 (3)	0.024 (3)	0.028 (3)	0.003 (2)	0.003 (2)	0.010 (2)
C45	0.018 (3)	0.018 (3)	0.032 (3)	0.004 (2)	0.007 (2)	0.003 (2)
C46	0.017 (3)	0.019 (3)	0.022 (2)	0.001 (2)	0.0033 (19)	0.002 (2)
C51	0.014 (2)	0.017 (2)	0.018 (2)	−0.0003 (19)	0.0034 (18)	0.0011 (19)
C52	0.017 (3)	0.014 (2)	0.020 (2)	0.0024 (19)	0.0056 (18)	0.0049 (19)
C53	0.022 (3)	0.019 (3)	0.016 (2)	0.001 (2)	0.0003 (19)	0.0002 (19)
C54	0.019 (3)	0.030 (3)	0.015 (2)	0.006 (2)	−0.0024 (18)	0.007 (2)
C55	0.019 (3)	0.020 (3)	0.028 (3)	0.006 (2)	0.000 (2)	0.008 (2)
C56	0.016 (3)	0.015 (2)	0.026 (3)	0.0032 (19)	0.0032 (19)	0.000 (2)
C61	0.016 (3)	0.014 (2)	0.020 (2)	0.0015 (19)	0.0030 (18)	−0.0011 (18)
C62	0.018 (3)	0.020 (3)	0.022 (3)	−0.001 (2)	0.0010 (19)	0.002 (2)
C63	0.019 (3)	0.019 (3)	0.036 (3)	−0.003 (2)	0.004 (2)	0.001 (2)
C64	0.033 (3)	0.023 (3)	0.028 (3)	0.002 (2)	0.013 (2)	0.009 (2)
C65	0.039 (3)	0.023 (3)	0.020 (3)	0.006 (2)	0.004 (2)	0.004 (2)
C66	0.020 (3)	0.019 (3)	0.026 (3)	0.002 (2)	0.000 (2)	−0.001 (2)
C71	0.013 (2)	0.017 (2)	0.010 (2)	0.0044 (19)	0.0055 (16)	0.0037 (17)
C72	0.013 (2)	0.019 (3)	0.027 (3)	0.002 (2)	0.0030 (19)	−0.002 (2)
C73	0.021 (3)	0.018 (3)	0.032 (3)	0.004 (2)	0.005 (2)	−0.001 (2)
C74	0.014 (3)	0.027 (3)	0.032 (3)	0.009 (2)	0.007 (2)	0.002 (2)
C75	0.012 (3)	0.031 (3)	0.024 (3)	0.000 (2)	0.0022 (19)	−0.001 (2)
C76	0.016 (3)	0.017 (2)	0.015 (2)	0.0004 (19)	0.0009 (18)	0.0052 (18)
C81	0.012 (2)	0.018 (2)	0.014 (2)	0.0036 (19)	0.0048 (17)	−0.0008 (18)
C82	0.021 (3)	0.019 (3)	0.021 (3)	0.002 (2)	0.0070 (19)	0.001 (2)
C83	0.020 (3)	0.029 (3)	0.020 (3)	0.001 (2)	0.002 (2)	−0.001 (2)
C84	0.022 (3)	0.033 (3)	0.018 (3)	0.009 (2)	0.001 (2)	0.004 (2)
C85	0.032 (3)	0.026 (3)	0.027 (3)	0.006 (2)	0.000 (2)	0.006 (2)
C86	0.025 (3)	0.022 (3)	0.020 (2)	0.003 (2)	−0.002 (2)	0.001 (2)
C91	0.059 (5)	0.014 (3)	0.053 (4)	0.002 (3)	−0.027 (3)	−0.003 (3)
C92	0.179 (13)	0.052 (5)	0.050 (5)	−0.014 (7)	−0.022 (6)	−0.007 (4)
C93	0.046 (5)	0.039 (4)	0.174 (9)	0.002 (4)	−0.038 (6)	0.031 (5)

### Geometric parameters (Å, º)

**Table:**

Au1—P1	2.3061 (12)	C42—H42	0.9500
Au1—P3	2.3102 (12)	C43—C44	1.389 (7)
Au1—Au2	2.9743 (2)	C43—H43	0.9500
Au2—P2	2.3082 (12)	C44—C45	1.393 (7)
Au2—P4	2.3130 (12)	C44—H44	0.9500
P1—C11	1.808 (5)	C45—C46	1.374 (7)
P1—C21	1.814 (4)	C45—H45	0.9500
P1—C1	1.826 (5)	C46—H46	0.9500
P2—C41	1.807 (5)	C51—C56	1.394 (7)
P2—C31	1.811 (5)	C51—C52	1.398 (7)
P2—C1	1.827 (5)	C52—C53	1.386 (7)
P3—C51	1.814 (5)	C52—H52	0.9500
P3—C61	1.825 (5)	C53—C54	1.383 (7)
P3—C2	1.835 (5)	C53—H53	0.9500
P4—C71	1.811 (5)	C54—C55	1.392 (7)
P4—C81	1.821 (5)	C54—H54	0.9500
P4—C2	1.828 (5)	C55—C56	1.386 (7)
O1—C91	1.205 (7)	C55—H55	0.9500
O1W—H1W	0.840 (10)	C56—H56	0.9500
O1W—H2W	0.841 (10)	C61—C66	1.388 (7)
C1—H1A	0.9900	C61—C62	1.392 (7)
C1—H1B	0.9900	C62—C63	1.372 (7)
C2—H2A	0.9899	C62—H62	0.9500
C2—H2B	0.9901	C63—C64	1.393 (8)
C11—C12	1.396 (6)	C63—H63	0.9500
C11—C16	1.396 (7)	C64—C65	1.366 (8)
C12—C13	1.383 (7)	C64—H64	0.9500
C12—H12	0.9500	C65—C66	1.402 (7)
C13—C14	1.379 (8)	C65—H65	0.9500
C13—H13	0.9500	C66—H66	0.9500
C14—C15	1.396 (8)	C71—C72	1.395 (7)
C14—H14	0.9500	C71—C76	1.411 (7)
C15—C16	1.381 (7)	C72—C73	1.391 (7)
C15—H15	0.9500	C72—H72	0.9500
C16—H16	0.9500	C73—C74	1.380 (7)
C21—C22	1.370 (7)	C73—H73	0.9500
C21—C26	1.406 (7)	C74—C75	1.391 (7)
C22—C23	1.391 (7)	C74—H74	0.9500
C22—H22	0.9500	C75—C76	1.381 (7)
C23—C24	1.375 (8)	C75—H75	0.9500
C23—H23	0.9500	C76—H76	0.9500
C24—C25	1.388 (8)	C81—C82	1.393 (7)
C24—H24	0.9500	C81—C86	1.393 (7)
C25—C26	1.381 (7)	C82—C83	1.390 (7)
C25—H25	0.9500	C82—H82	0.9500
C26—H26	0.9500	C83—C84	1.391 (7)
C31—C32	1.389 (7)	C83—H83	0.9500
C31—C36	1.393 (7)	C84—C85	1.366 (7)
C32—C33	1.376 (7)	C84—H84	0.9500
C32—H32	0.9500	C85—C86	1.393 (7)
C33—C34	1.396 (8)	C85—H85	0.9500
C33—H33	0.9500	C86—H86	0.9500
C34—C35	1.380 (8)	C91—C92	1.475 (11)
C34—H34	0.9500	C91—C93	1.488 (12)
C35—C36	1.387 (7)	C92—H92A	0.9800
C35—H35	0.9500	C92—H92B	0.9800
C36—H36	0.9500	C92—H92C	0.9800
C41—C42	1.393 (6)	C93—H93A	0.9800
C41—C46	1.406 (7)	C93—H93B	0.9800
C42—C43	1.374 (7)	C93—H93C	0.9800
			
P1—Au1—P3	173.24 (4)	C43—C42—H42	119.6
P1—Au1—Au2	91.96 (3)	C41—C42—H42	119.6
P3—Au1—Au2	91.83 (3)	C42—C43—C44	120.3 (5)
P2—Au2—P4	170.04 (4)	C42—C43—H43	119.8
P2—Au2—Au1	90.59 (3)	C44—C43—H43	119.8
P4—Au2—Au1	90.86 (3)	C43—C44—C45	119.3 (5)
C11—P1—C21	103.6 (2)	C43—C44—H44	120.4
C11—P1—C1	107.3 (2)	C45—C44—H44	120.4
C21—P1—C1	103.1 (2)	C46—C45—C44	120.6 (5)
C11—P1—Au1	115.57 (15)	C46—C45—H45	119.7
C21—P1—Au1	114.70 (15)	C44—C45—H45	119.7
C1—P1—Au1	111.43 (16)	C45—C46—C41	120.2 (4)
C41—P2—C31	106.7 (2)	C45—C46—H46	119.9
C41—P2—C1	107.8 (2)	C41—C46—H46	119.9
C31—P2—C1	101.9 (2)	C56—C51—C52	119.4 (4)
C41—P2—Au2	112.26 (16)	C56—C51—P3	118.4 (4)
C31—P2—Au2	116.58 (15)	C52—C51—P3	122.1 (4)
C1—P2—Au2	110.82 (16)	C53—C52—C51	120.2 (4)
C51—P3—C61	107.0 (2)	C53—C52—H52	120.2
C51—P3—C2	109.0 (2)	C51—C52—H52	119.6
C61—P3—C2	102.6 (2)	C54—C53—C52	120.2 (4)
C51—P3—Au1	113.79 (16)	C54—C53—H53	119.9
C61—P3—Au1	112.42 (15)	C52—C53—H53	119.9
C2—P3—Au1	111.33 (16)	C53—C54—C55	119.8 (4)
C71—P4—C81	106.8 (2)	C53—C54—H54	120.1
C71—P4—C2	104.0 (2)	C55—C54—H54	120.1
C81—P4—C2	106.6 (2)	C56—C55—C54	120.4 (5)
C71—P4—Au2	113.82 (16)	C56—C55—H55	119.8
C81—P4—Au2	112.80 (16)	C54—C55—H55	119.8
C2—P4—Au2	112.24 (16)	C55—C56—C51	119.9 (5)
H1W—O1W—H2W	107 (5)	C55—C56—H56	120.0
P1—C1—P2	115.1 (3)	C51—C56—H56	120.0
P1—C1—H1A	108.6	C66—C61—C62	119.3 (4)
P2—C1—H1A	108.6	C66—C61—P3	117.1 (4)
P1—C1—H1B	108.4	C62—C61—P3	123.5 (4)
P2—C1—H1B	108.4	C63—C62—C61	120.8 (5)
H1A—C1—H1B	107.5	C63—C62—H62	119.6
P4—C2—P3	114.6 (3)	C61—C62—H62	119.6
P4—C2—H2A	108.6	C62—C63—C64	119.9 (5)
P3—C2—H2A	108.5	C62—C63—H63	120.0
P4—C2—H2B	108.7	C64—C63—H63	120.0
P3—C2—H2B	108.6	C65—C64—C63	120.0 (5)
H2A—C2—H2B	107.6	C65—C64—H64	120.0
C12—C11—C16	118.8 (4)	C63—C64—H64	120.0
C12—C11—P1	120.8 (4)	C64—C65—C66	120.5 (5)
C16—C11—P1	120.3 (4)	C64—C65—H65	119.7
C13—C12—C11	120.3 (5)	C66—C65—H65	119.7
C13—C12—H12	119.9	C61—C66—C65	119.5 (5)
C11—C12—H12	119.9	C61—C66—H66	120.3
C14—C13—C12	120.3 (5)	C65—C66—H66	120.3
C14—C13—H13	119.9	C72—C71—C76	118.9 (4)
C12—C13—H13	119.9	C72—C71—P4	123.3 (4)
C13—C14—C15	120.4 (5)	C76—C71—P4	117.8 (4)
C13—C14—H14	119.8	C73—C72—C71	119.8 (5)
C15—C14—H14	119.8	C73—C72—H72	120.1
C16—C15—C14	119.2 (5)	C71—C72—H72	120.1
C16—C15—H15	120.4	C74—C73—C72	121.0 (5)
C14—C15—H15	120.4	C74—C73—H73	119.5
C15—C16—C11	121.1 (5)	C72—C73—H73	119.5
C15—C16—H16	119.5	C73—C74—C75	119.7 (5)
C11—C16—H16	119.5	C73—C74—H74	120.2
C22—C21—C26	119.7 (4)	C75—C74—H74	120.2
C22—C21—P1	123.6 (4)	C76—C75—C74	120.2 (5)
C26—C21—P1	116.7 (3)	C76—C75—H75	119.9
C21—C22—C23	119.8 (5)	C74—C75—H75	119.9
C21—C22—H22	120.1	C75—C76—C71	120.4 (5)
C23—C22—H22	120.1	C75—C76—H76	119.8
C24—C23—C22	120.9 (5)	C71—C76—H76	119.8
C24—C23—H23	119.5	C82—C81—C86	119.4 (4)
C22—C23—H23	119.5	C82—C81—P4	122.2 (4)
C23—C24—C25	119.5 (5)	C86—C81—P4	118.4 (4)
C23—C24—H24	120.3	C83—C82—C81	120.6 (5)
C25—C24—H24	120.3	C83—C82—H82	119.7
C26—C25—C24	120.2 (5)	C81—C82—H82	119.7
C26—C25—H25	119.9	C82—C83—C84	119.3 (5)
C24—C25—H25	119.9	C82—C83—H83	120.3
C25—C26—C21	119.9 (5)	C84—C83—H83	120.3
C25—C26—H26	120.1	C85—C84—C83	120.3 (5)
C21—C26—H26	120.1	C85—C84—H84	119.8
C32—C31—C36	120.0 (5)	C83—C84—H84	119.8
C32—C31—P2	118.2 (4)	C84—C85—C86	121.0 (5)
C36—C31—P2	121.8 (4)	C84—C85—H85	119.5
C33—C32—C31	120.2 (5)	C86—C85—H85	119.5
C33—C32—H32	119.8	C85—C86—C81	119.4 (5)
C31—C32—H32	120.0	C85—C86—H86	120.3
C32—C33—C34	120.5 (5)	C81—C86—H86	120.3
C32—C33—H33	119.8	O1—C91—C92	121.4 (8)
C34—C33—H33	119.8	O1—C91—C93	120.2 (8)
C35—C34—C33	118.9 (5)	C92—C91—C93	118.3 (7)
C35—C34—H34	120.6	C91—C92—H92A	109.5
C33—C34—H34	120.6	C91—C92—H92B	109.5
C34—C35—C36	121.4 (5)	H92A—C92—H92B	109.5
C34—C35—H35	119.3	C91—C92—H92C	109.5
C36—C35—H35	119.3	H92A—C92—H92C	109.5
C35—C36—C31	119.0 (5)	H92B—C92—H92C	109.5
C35—C36—H36	120.5	C91—C93—H93A	109.5
C31—C36—H36	120.5	C91—C93—H93B	109.5
C42—C41—C46	118.7 (4)	H93A—C93—H93B	109.5
C42—C41—P2	118.5 (4)	C91—C93—H93C	109.5
C46—C41—P2	122.8 (4)	H93A—C93—H93C	109.5
C43—C42—C41	120.8 (4)	H93B—C93—H93C	109.5
			
C11—P1—C1—P2	80.8 (3)	C41—C42—C43—C44	1.9 (8)
C21—P1—C1—P2	−170.2 (3)	C42—C43—C44—C45	−3.3 (8)
Au1—P1—C1—P2	−46.6 (3)	C43—C44—C45—C46	1.6 (8)
C41—P2—C1—P1	−71.9 (3)	C44—C45—C46—C41	1.5 (8)
C31—P2—C1—P1	176.0 (2)	C42—C41—C46—C45	−2.9 (7)
Au2—P2—C1—P1	51.3 (3)	P2—C41—C46—C45	174.6 (4)
C71—P4—C2—P3	−171.5 (2)	C61—P3—C51—C56	−113.0 (4)
C81—P4—C2—P3	75.9 (3)	C2—P3—C51—C56	136.7 (4)
Au2—P4—C2—P3	−48.1 (3)	Au1—P3—C51—C56	11.8 (4)
C51—P3—C2—P4	−78.5 (3)	C61—P3—C51—C52	64.5 (4)
C61—P3—C2—P4	168.3 (3)	C2—P3—C51—C52	−45.8 (5)
Au1—P3—C2—P4	47.8 (3)	Au1—P3—C51—C52	−170.7 (3)
C21—P1—C11—C12	−40.5 (4)	C56—C51—C52—C53	1.0 (7)
C1—P1—C11—C12	68.2 (4)	P3—C51—C52—C53	−176.4 (4)
Au1—P1—C11—C12	−166.8 (3)	C51—C52—C53—C54	−1.5 (7)
C21—P1—C11—C16	140.7 (4)	C52—C53—C54—C55	0.8 (8)
C1—P1—C11—C16	−110.6 (4)	C53—C54—C55—C56	0.5 (8)
Au1—P1—C11—C16	14.4 (4)	C54—C55—C56—C51	−1.0 (8)
C16—C11—C12—C13	1.4 (7)	C52—C51—C56—C55	0.2 (7)
P1—C11—C12—C13	−177.4 (4)	P3—C51—C56—C55	177.8 (4)
C11—C12—C13—C14	−0.4 (7)	C51—P3—C61—C66	165.7 (4)
C12—C13—C14—C15	−1.0 (8)	C2—P3—C61—C66	−79.7 (4)
C13—C14—C15—C16	1.3 (8)	Au1—P3—C61—C66	40.0 (4)
C14—C15—C16—C11	−0.2 (8)	C51—P3—C61—C62	−11.5 (5)
C12—C11—C16—C15	−1.1 (7)	C2—P3—C61—C62	103.1 (4)
P1—C11—C16—C15	177.7 (4)	Au1—P3—C61—C62	−137.2 (4)
C11—P1—C21—C22	121.8 (4)	C66—C61—C62—C63	−0.3 (7)
C1—P1—C21—C22	10.0 (5)	P3—C61—C62—C63	176.8 (4)
Au1—P1—C21—C22	−111.3 (4)	C61—C62—C63—C64	0.0 (8)
C11—P1—C21—C26	−57.9 (4)	C62—C63—C64—C65	0.6 (8)
C1—P1—C21—C26	−169.7 (4)	C63—C64—C65—C66	−0.8 (8)
Au1—P1—C21—C26	69.0 (4)	C62—C61—C66—C65	0.1 (7)
C26—C21—C22—C23	−0.1 (7)	P3—C61—C66—C65	−177.2 (4)
P1—C21—C22—C23	−179.8 (4)	C64—C65—C66—C61	0.4 (8)
C21—C22—C23—C24	0.0 (8)	C81—P4—C71—C72	72.3 (4)
C22—C23—C24—C25	−0.1 (9)	C2—P4—C71—C72	−40.1 (4)
C23—C24—C25—C26	0.4 (8)	Au2—P4—C71—C72	−162.6 (3)
C24—C25—C26—C21	−0.4 (8)	C81—P4—C71—C76	−105.6 (4)
C22—C21—C26—C25	0.3 (7)	C2—P4—C71—C76	142.0 (3)
P1—C21—C26—C25	180.0 (4)	Au2—P4—C71—C76	19.6 (4)
C41—P2—C31—C32	−178.9 (4)	C76—C71—C72—C73	1.0 (7)
C1—P2—C31—C32	−66.0 (4)	P4—C71—C72—C73	−176.8 (4)
Au2—P2—C31—C32	54.7 (4)	C71—C72—C73—C74	−1.1 (7)
C41—P2—C31—C36	3.4 (4)	C72—C73—C74—C75	0.4 (8)
C1—P2—C31—C36	116.3 (4)	C73—C74—C75—C76	0.3 (8)
Au2—P2—C31—C36	−122.9 (4)	C74—C75—C76—C71	−0.4 (7)
C36—C31—C32—C33	0.7 (8)	C72—C71—C76—C75	−0.3 (7)
P2—C31—C32—C33	−177.0 (4)	P4—C71—C76—C75	177.7 (4)
C31—C32—C33—C34	0.6 (8)	C71—P4—C81—C82	−64.7 (4)
C32—C33—C34—C35	−1.4 (8)	C2—P4—C81—C82	45.9 (4)
C33—C34—C35—C36	0.9 (8)	Au2—P4—C81—C82	169.5 (3)
C34—C35—C36—C31	0.3 (8)	C71—P4—C81—C86	114.4 (4)
C32—C31—C36—C35	−1.1 (7)	C2—P4—C81—C86	−134.9 (4)
P2—C31—C36—C35	176.5 (4)	Au2—P4—C81—C86	−11.3 (4)
C31—P2—C41—C42	−101.7 (4)	C86—C81—C82—C83	1.5 (7)
C1—P2—C41—C42	149.5 (4)	P4—C81—C82—C83	−179.3 (4)
Au2—P2—C41—C42	27.2 (4)	C81—C82—C83—C84	−1.4 (7)
C31—P2—C41—C46	80.8 (4)	C82—C83—C84—C85	0.6 (8)
C1—P2—C41—C46	−28.0 (5)	C83—C84—C85—C86	−0.1 (8)
Au2—P2—C41—C46	−150.3 (4)	C84—C85—C86—C81	0.2 (8)
C46—C41—C42—C43	1.2 (7)	C82—C81—C86—C85	−1.0 (7)
P2—C41—C42—C43	−176.4 (4)	P4—C81—C86—C85	179.9 (4)

### Hydrogen-bond geometry (Å, º)

Cg1–Cg3 are the ring centroids of the C11–C16, C71–C76 and C51–C56 benzene rings, respectively.

**Table:**

*D*—H···*A*	*D*—H	H···*A*	*D*···*A*	*D*—H···*A*
O1*W*—H1*W*···Cl2	0.84 (2)	2.39 (2)	3.217 (5)	168 (6)
O1*W*—H2*W*···Cl2^i^	0.84 (3)	2.37 (3)	3.200 (5)	173 (3)
C1—H1*B*···O1^ii^	0.99	2.35	3.310 (7)	164
C2—H2*B*···Cl2^iii^	0.99	2.51	3.487 (5)	168
C12—H12···O1^ii^	0.95	2.57	3.249 (7)	129
C22—H22···Cl1^ii^	0.95	2.74	3.580 (6)	148
C44—H44···Cl2^iv^	0.95	2.73	3.424 (5)	130
C52—H52···Cl2^iii^	0.95	2.68	3.616 (5)	169
C82—H82···Cl2^iii^	0.95	2.81	3.723 (5)	161
C34—H34···*Cg*1^v^	0.95	2.82	3.542 (6)	133
C43—H43···*Cg*2^vi^	0.95	2.74	3.574 (5)	147
C75—H75···*Cg*3^v^	0.95	2.83	3.619 (5)	142

Symmetry codes: (i) −*x*+1, −*y*+1, −*z*; (ii) −*x*, −*y*, −*z*+1; (iii) −*x*+1, −*y*+1, −*z*+1; (iv) −*x*+1, −*y*, −*z*+1; (v) *x*−1, *y*, *z*; (vi) −*x*, −*y*, −*z*+2.
